# Prevalence Estimates of Symptom Feigning and Malingering in Spain

**DOI:** 10.1007/s12207-022-09458-w

**Published:** 2022-07-26

**Authors:** Esteban Puente-López, David Pina, Reyes López-López, Héctor González Ordi, Irena Bošković, Thomas Merten

**Affiliations:** 1 Universidad Nebrija, Madrid, Spain; 2 Universidad de Murcia, Murcia, Spain; 3 Universidad Complutense de Madrid, Madrid, Spain; 4 Erasmus University Rotterdam, Rotterdam, Netherlands; 5 Vivantes Klinikum Im Friedrichshain, Berlin, Germany

**Keywords:** Feigning, Malingering, Illness presentation, Prevalence, Survey

## Abstract

Symptom feigning and malingering should be evaluated in forensic contexts due to their important socio-economic consequences. Despite this, to date, there is little research in Spain that evaluates its prevalence. The aim of this study was to investigate this issue using the perception of the general population, students, and professionals of medicine and forensic psychology. Three adapted questionnaires were applied to a total of 1003 participants (61.5% women) from 5 different groups. Approximately two-thirds of participants reported knowing someone who feigned symptoms, and one-third disclosed feigning symptoms themselves in the past. Headache/migraine, neck pain, and anxious–depressive symptoms were the most commonly chosen. Experts in psychology and forensic medicine estimated a prevalence of 20 to 40% of non-credible symptom presentations in their work settings and reported not having sufficient means to assess the distorted presentation of symptoms with certainty. Professionals and laypersons alike acknowledge that non-credible symptom presentations (like feigning or malingering) are relevant in Spain and occur at a non-trivial rate, which compares with estimates in other parts of the world.

Assessments made in both clinical and forensic settings depend, to a large extent, on the symptom presentation of the person to be evaluated, their openness and accuracy in responding, and their willingness to make a sincere and sustained effort (Merckelbach et al., 2019). Therefore, the practitioner must consider the possibility that patients may be deceitful in their symptom presentations due to goals or motives unrelated to the diagnosis or condition (Merten & Merckelbach, 2020). In a clinical context, a patient may obtain gains associated with having a disease (affective benefits) by acquiring the status of being ill. In forensic contexts, having certain diagnosis may help to gain legal or financial benefits, such as limited criminal liability or financial compensation for personal injury or disability. In many cases, both primary (internal) and secondary (external) benefits occur simultaneously (González-Ordi et al., 2012; Merten & Merckelbach, 2020). When symptom deception is followed by known external gains, it is called malingering. However, when the type of motive driving such behavior is unknown, the term feigning is preferred (Rogers & Bender, 2018). Feigning includes both malingering and other forms of factitious illness presentations.

A considerable amount of research focuses on establishing prevalence estimates of feigning across cultures and reference contexts (e.g., Dandachi-FitzGerald et al., 2020; Santamaría et al., 2013; Schroeder et al., 2021). Researchers in the field of symptom validity assessment make exceptional efforts to establish the prevalence of feigning across cultures, but the available estimates of the prevalence of feigning/malingering differ significantly and it is difficult to arrive at a precise range due to the great heterogeneity of published studies (Merten & Merckelbach, 2020). For example, Mittenberg et al. (2002) found that, depending on the setting (i.e., criminal, civil or medical), feigning was suspected in approximately 7 to 31% of neuropsychological assessment cases. Greve et al. (2009) examined the prevalence in 508 chronic pain patients seeking compensation in North America, finding a base rate of 32.5 to 35%. Similarly, Chafetz (2011) examined the performance of 161 social security disability claimants, finding that 38.5% were classified as either probable or definite malingerers (15% were classified as definite malingerers). However, these base rates are lower than other base rate estimates for North America, such as the 46% in the 242 social security disability claimants of Schroeder et al. (2021), and considerably higher than the 9.9% UK base rate estimated by expert psychologists (Cartwright et al., 2019), the 5–10% base rate of Australian psychologists doing medicolegal work (Yoxall et al., 2010), and the 13% among Australian neuropsychologists (Sullivan et al., 2006). In Spain, Santamaría et al. (2013) found that professionals estimated the prevalence rate to be close to 50% in cases of chronic pain disorders, with whiplash injury having the highest incidence, followed by fibromyalgia, chronic cervicalgia, chronic low back pain, and affective and emotional disorders. The professionals also perceived a greater frequency of feigning in women, middle-aged people, with low income and unstimulating jobs. They judged that the most commonly used mechanism of distorted symptom presentation was the perpetuation of symptoms claims after improvement.

Recently, Dandachi-FitzGerald et al. (2020) asked a large number of participants about the symptom behavior of third parties (called “Ten Questions About Feigning,” TQAF), and their data revealed that 59% of the participants indicated that they knew a probable feigner, and 34% knew a person who had admitted to them that they had feigned symptoms themselves. The symptoms most often feigned were very common, like headache or fever, and the most important reasons for feigning were to obtain an authorized leave from work, to excuse a failure, and to seek attention from others.

These results, among others, indicate that symptom deception (feigning) occurs on a non-trivial scale and that further research into its prevalence across cultures is very important. For this reason, we aimed at replicating the research of Dandachi-FitzGerald et al. (2020) in Spain and extend it to several additional groups of interest, such as psychology students, psychologists, and medicolegal professionals. Our main objective was to assess the perception of symptom feigning among the general population and students as well as among professionals working in this field. In this survey, we specifically wanted to assess attitudes and beliefs about feigning of three non-expert samples: general population, university students, and psychology students. This was conceived to provide information on how common feigning is in the everyday life. Yet, as these findings cannot be generalized to forensic contexts, due to the differences in the incentives and consequences of failed feigning, we also asked two samples of forensic experts (forensic doctors and psychologists) to estimate the prevalence of feigning in their practice and we investigated experts’ beliefs about feigning and methods used for its assessment.

## Method

### Participants

The total sample consisted of 1003 participants, of whom 617 were female (61.5%). They were all inhabitants of Spain or had Spanish as their native language. The sample comprised the following subsamples: unselected university students (*n* = 415; mean age = 21.2; *SD* = 6.3), fourth-year psychology students (*n* = 122; mean age = 22.5; *SD* = 4.5), general population (*n* = 378; mean age = 33.6, *SD* = 11.3; 37% of them have a university degree and an additional 47% have a high school education), psychologists specialized in forensic psychology (*n* = 40, mean age = 32.4, *SD* = 13.3), and physicians specialized in forensic/legal medicine *(n* = 48, mean age = 39.5, *SD* = 15.5). The response rate of the unselected group of students was 59% (*n* contacted = 700), whereas the response rate of forensic professionals was 38.8% for psychologists (*n* contacted = 103) and 50.5% for physicians (*n* contacted = 95). The response rate of psychology students was unknown because they were not contacted directly, but through their university.

### Instruments

This project included three surveys based on the *ten questions about feigning* formulated by Dandachi-FitzGerald et al. (2020). The first survey (Annex 1), administered to the general population and students, is a Spanish adaptation of those ten questions, seven of them in multiple-choice format and three open-ended. The second survey (Annex 2), conducted with fourth-year psychology students, consisted of 15 questions, ten from the original list and five additional questions aimed at evaluating the degree of training obtained by psychology students. The third survey (Annex 3) consisted of 17 questions, ten from the original list and seven additional questions that were adapted from a survey by Santamaría et al. (2013), adjusting the contents and the wording to the present study. The questions were initially designed by one of the authors and were reviewed by the rest until a consensus was reached.

### Procedure

The online survey participants were included using convenience sampling. The procedure was divided into three blocks. In the first block, university students were contacted through the virtual survey system of the university of one of the authors. After completing the survey, they were asked to forward it to an acquaintance who was not a student. This was done in order to collect data from the general public. In the second block, the directors of the psychology degree of the Spanish public universities were contacted and asked to forward the survey to fourth-year psychology students. At the end of the survey, they were also asked to send the link to the survey to an acquaintance who was not a student. In the third block, medical and forensic psychology practitioners were contacted. For this purpose, one of the authors conducted a search for the experts recommended from the websites of Spanish universities and professional associations. In addition, a Google search was conducted, using the terms “forensic doctor/psychologist Spain” and “forensic doctor/psychologist (autonomous community).”

This study was approved by the Research Ethics Committee of one of the authors’ University. No incentive was offered for participation. However, participants were told that, if they wished to know the results of the study, they could contact one of the authors and receive a report of the main findings.

## Results

### Prevalence Estimates

Of the total sample (*n* = 1003), 625 participants (62.3%) indicated that they knew one or more people who had feigned or were currently feigning symptoms or an illness. Of those 625 participants, 40 belonged to the forensic psychologists group (6.4% of *n* = 625), 48 to the forensic physicians group (7.6%), 231 to the general population group (36.9%), 71 to the psychology students group (11.3%), and the remaining 235 to the students group (37.6%). When asked how they knew that the person was feigning, 357 indicated that the person had told them (57.12%), 154 because someone else told them (24.6%), 92 knew it by intuition (14.7%), and 18 concluded that they were feigning because the symptom presentation was not credible (2.3%).

Of the 1003 participants, 341 (33.9%) indicated that they themselves had feigned symptoms or an illness at some time in the past. Of those, 197 belonged to the general population group (19.6%), 18 were psychology students (19.6%), 124 were students (12.3%), and 2 were forensic psychologists (0.1%).

### Feigned Symptoms and Motives

Table 1 shows the four most frequently feigned symptoms as reported by the participants of each group. As can be seen at the top of Table 1, the symptoms most frequently mentioned were migraines/headache, neck pain, and depressive and anxiety symptoms.

**Table 1:** Reported symptoms most frequently feigned by the participants of the subgroups

Group	*N*	Percentage (of total sample)	95% confidence interval	Percentage (of subgroup)
*Total sample*	*1003*	*–*	*–*	*–*
Migraines/headaches	176	17%	15–20%	*–*
Cervical damage	159	15.8%	13–18%	*–*
Depressive symptoms	157	15.6%	13–18%	*–*
Anxiety/stress	125	12.4%	10–14%	*–*
*Students*	*415*			
Migraines/headaches	154	15.4%	13–17%	37.1%
Cold/flu	84	8.4%	6–10%	20.2%
Depressive symptoms	65	6.5%	5–8%	15.7%
Anxiety/stress	42	4.2%	3–5%	10.1%
*General population*	*378*			
Cervical damage	121	12.1%	10–14%	32.0%
Pain (general)	87	8.7%	7–10%	23.0%
Anxiety/stress	54	5.4%	4–6%	14.3%
Depressive symptoms	36	3.6%	2–4%	9.5%
*Psychology students*	*122*			
Depressive symptoms	44	4.4%	3–5%	36.1%
Memory problems	29	2.9%	1–4%	23.8%
Anxiety/stress	24	2.4%	1–3%	19.7%
Migraines/headache	15	1.5%	0.8–2%	12.3%
*Forensic and legal medicine physicians*	*48*	*–*	*–*	–
Cervical damage	24	2.4%	1–3%	50.0%
Pain (general)	8	0.8%	0.3–1%	16.7%
Low back pain	5	0.5%	0.1–1%	10.4%
Depressive symptoms	4	0.4%	0.1–1%	8.3%
*Forensic psychologists*	*40*	*–*	*–*	–
Cervical damage	14	1.4%	0.7–2%	35.0%
Depressive symptoms	8	0.8%	0.3–1%	20.0%
Migraines/headache	7	0.7%	0.2–1%	17.5%
Anxiety	5	0.5%	0.1–1%	12.5%

Regarding the motivation for feigning symptoms or an illness, Table 2 shows the three main incentives mentioned in each group. In the general population and in the students’ samples, sick leave from work or education was most frequently reported (42.4% and 38.1%, respectively). In the sample of psychology students and forensic psychologists, it was the gain of social security benefits (44.3% and 62.5%, respectively). The physicians considered the gain of economic benefits (provided by private medical insurance companies) to be the main cause for feigning an illness or disability. In the total sample, the reasons stated for feigning were sick leave (36.3%), social security benefits (20.6%), disability or sick pay provided by a private insurance company (15%), extended vacation/extra days off work (11.9%), financial compensation (6.3%), receiving care from others (4.3%), securing benefits in a legal proceeding (2.9%), excusing a mistake or failure (1.7%), and residence permit in Spain (0.9%).

**Table 2:** Three most frequent reasons for feigning (for the total sample and by group)

Group	*N*	Percentage (of total sample)	95% confidence interval	Percentage (of subgroup)
*Total sample*	*1003*			
Sick leave from work or education	364	36.3%	0.33–0.39%	–
Social security benefits	207	20.6%		–
Disability or sick pay provided by a private insurance company	150	15.0%	0.18–0.23%	–
*Students*	*415*	–	–	–
Sick leave from work or education	176	17.5%	0.15–0.20%	42.4%
Extension of vacation/extra days off work	85	8.5%	0.6–0.10%	20.5%
Disability or sick pay provided by a private insurance company	72	7.2%	0.5–0.8%	17.3%
*General sample*	*378*			
Sick leave from work or education	144	14.4%	0.12–0.16%	38.1%
Social security benefits	110	11.0%	0.9–0.13%	29.1%
Disability or sick pay provided by a private insurance company	54	5.4%	0.4–0.6%	14.3%
*Psychology students*	*122*			
Social security benefits	54	5.4%	0.4–0.6%	44.3%
Sick leave from work or education	32	3.2%	0.2–0.4%	26.2%
Securing benefits in a legal proceeding	11	1.1%	0.05–0.1%	9.0%
*Forensic and legal medicine physicians*	*48*			
Disability or sick pay provided by a private insurance company	19	1.9%	0.1–0.2%	39.6%
Social security benefits	18	1.8%	0.1–0.2%	37.5%
Sick leave from work or education	8	0.8%	0.03–1%	16.7%
*Forensic psychologists*	*40*	–	–	–
Social security benefits	25	2.5%	0.1–0.3%	62.5%
Disability or sick pay provided by a private insurance company	5	0.5%	0.01–0.1%	12.5%
Sick leave from work or education	4	0.4%	0.01–0.1%	10.0%

### Symptoms Feigned by Participants and Reasons for Feigning Them

Participants were asked which symptoms or illnesses they would choose to feign. Participants indicated a wide variety of symptomatology, with headache or migraines being the most frequent. A total of 342 participants (34.1%) stated that they would feign these symptoms, followed by other pain-related symptoms (325 participants, 32.4%). This category was broken down into 115 participants indicating neck pain (11.4%), 73 general pain (7.2%), 61 low back pain (6.0%), 49 stomach pain (4.8%), and 27 fibromyalgia-type pain (2.6%). In third place, participants indicated anxiety and depressive symptoms, chosen by 110 (10.9%) and 97 (9.6%) participants, respectively. Sixty-two participants indicated common cold/flu (6.1%), 37 indicated memory problems (3.6%), 8 indicated problems related to intelligence and comprehension (0.7%), 7 indicated psychotic-like symptomatology (0.6%), 7 indicated motor problems (0.6%), 6 indicated COVID-19 symptoms (0.5%), and 2 reported respiratory symptoms (0.1%).

When asked for reasons why they had chosen their preferred symptoms (with multiple answers allowed), 557 indicated that it was easy to feign (55.5%), 372 said they had previously experienced these symptoms (37.0%), 221 said they knew someone who had experienced these symptoms (22.0%), 111 indicated that they were easy to feign over an extended period of time (11.0%), and 51 responded that these symptoms would make a strong impression on others (5.0%).

Participants were also asked which symptoms or illnesses they would avoid feigning. The responses were cancer in 451 cases (44.9%), followed by diseases/illnesses with possible physical evidence (382; 38.0%), such as a bone fracture or muscle injury, COVID-19 infection (110; 10.9%), and various diseases with biological markers such as acquired immunodeficiency syndrome, hepatitis, or lung problems (60; 5.9%). Regarding the reasons for marking the above symptoms (with the possibility of multiple responses), 678 considered it morally unacceptable to feign them (67.5%), 522 considered them difficult to feign (52.0%), 518 estimated the probability of detection to be very high (51.6%), and 336 considered it difficult to feign them convincingly (33.4%). As an additional free response, 185 participants (18.4%) wrote that it was “virtually” impossible to feign the symptoms in question due to the existence of very objective biological/physical markers.

### The Knowledge of Psychology Students

The results obtained in the five questions prepared specifically to assess the subject knowledge of the group of psychology students are shown in Table 3. All the participants were involved in legal, forensic, and/or criminal psychology subjects; 89 of them (72.9%) received university training on malingering. As can be seen, most participants (86.1%) indicated that such training was brief and part of a broader subject.

**Table 3:** Items related to symptom validity training and malingering (psychology undergraduates, *N* = 122)

	*n*	%
*11. You have taken a course on legal, forensic and/or criminal psychology*		
Yes	122	100%
*12. Subject matter on which training has been received in the psychology degree – more than one answer possible*		
Malingered symptom presentation	89	73.0%
Exaggeration of symptoms / feigning	36	29.5%
Distorted symptom presentation	10	8.2%
Symptom validity	8	6.6%
Distortion in response styles	2	1.6%
*12.1. You have taken external/non-degree training in psychology related to the subject*		
Yes	17	13.9%
*12.2. Extension of the training received*		
Approximately one-third of the lesson of a subject	105	86.1%
Half of the lesson of a subject	9	7.4%
A complete lesson of a subject	8	6.6%
*13. Strategies/instruments for detection of distorted presentation of symptoms/malingering (more than one answer allowed)*		
Structured Inventory of Malingered Symptomatology	58	47.5%
Clinical Forensic Interview by Arce and Fariña	49	40.2%
Global Evaluation System	48	39.3%
Test of Memory Malingering	35	28.7%
Validity scales of the Minnesota Multiphasic Personality Inventory family	10	8.2%
Validity scales of the Millon Clinical Multiaxial Inventory	8	6.6%
Structured Interview of Reported Symptoms	8	6.6%
Validity scales of the Personality Assessment Inventory	4	3.3%
*14. How necessary it is for the psychologist to rule out distorted symptom presentation/malingering*		
2. Somewhat important	52	42.6%
3. Important	36	29.5%
4. Very important	21	17.2%
5. Fundamental (must be done)	7	5.7%
1. Not important	6	4.9%
*15. Degree of training received in the subject matter in their university studies*		
2. Insufficient	55	45.1%
3. Sufficient	47	38.5%
4. Very sufficient	10	8.2%
1. Very insufficient	10	8.2%

### The Experience of Forensic Professionals

For the group of forensic psychology professionals, seven additional questions were included about their experience in the field (Table 4). More than half of them (60%) estimated that between 20 and 40% of their cases present a risk of feigning/malingering, pointing out depressive symptomatology as the most feigned symptom. Leave of absence from work or education was perceived as the most common reason for feigning/malingering (40%). Psychometric screening instruments to assess feigning and diagnostic psychometric instruments with embedded validity scales were the most commonly used methods to detect the risk of feigning/malingering (80% and 62.5% respectively). A vast majority of forensic psychologists also indicated that inconsistencies between clinical presentation and findings (80%), as well as disproportionate or magnified expression of symptoms (72.5%), are the data that raise their suspicion of feigning/malingering the most. Approximately two-thirds of forensic psychologists (62.5%) think that they do not have sufficient means to detect feigning/malingering with an appropriate level of certainty, and the vast majority (82.5%) indicate that it would be of interest to develop new methods, techniques, or tools to detect feigning/malingering.

**Table 4:** Experience of forensic psychologists (*N* = 40)

	*N*	%
*11. Risk of feigning/malingering in your daily professional practice – annual estimate*		
Moderate frequency (between 20 and 40% of your cases)	24	60.0%
High frequency (above 40%)	11	27.5%
Frequency lower than 20% of cases	5	12.0%
*12. Symptoms or diseases most commonly feigned by YOUR OWN patients/clients*		
Depressive symptoms	22	55.0%
Memory problems	8	20.0%
Anxiety in general	5	12.5%
Post–traumatic stress	4	10.0%
Psychotic symptoms	1	2.5%
*13. Most common reasons for YOUR patients to feign*		
Leave of absence from work or education	15	40.0%
Social security benefits	13	32.5%
Securing benefits in a legal proceeding	7	17.5%
Disability or sick pay provided by a private insurance company	2	5.0%
To receive care from others	2	5.0%
To excuse a mistake or failure	1	2.5%
*14. Method(s) used to detect the risk of feigning/malingering – possibility to choose more than one response*		
Psychometric screening instruments to assess feigning	32	80.0%
Diagnostic psychometric instruments with embedded validity scales	25	62.5%
Clinical impression complementary to the use of specialized psychometric instruments	17	42.5%
Standardized interviews for malingering screening	12	30.0%
*15. Data that raise suspicion of feigning/malingering*		
Inconsistency between clinical presentation and findings	32	80.0%
Disproportionate or magnified symptom expression	29	72.5%
Hostility, lack of collaboration, defensiveness	21	52.5%
Obtaining an external or internal gain	20	50.0%
Excessively detailed presentation	18	45.0%
Lack of response to treatment	12	30.0%
*Adequacy of means to assess the risk of feigning/malingering (question 16)*		
I have the means to detect it, but I do not believe that they are sufficient to detect it with an appropriate level of certainty	25	62.5%
I have few means to detect it with certainty	8	20%
I have sufficient means to detect it reliably	7	17.5%
*Would it be of interest to develop new methods, techniques or tools to assess the risk of feigning/malingering? (question 17)*		
Yes, I think it would be necessary for my discipline	33	82.5%

As with the previous group, a third survey was conducted for the group of medical professionals, with seven additional questions on their experience with the subject. Table 5 shows a summary of the main results obtained in these questions. About half of the group (52.1%) estimated that between 20 and 40% of their cases present a risk of feigning/malingering, pointing out cervical damage (including mainly whiplash related injuries) as the most feigned symptom. Disability or sick pay provided by a private insurance company is perceived as the most common reason for feigning/malingering (35.4%). The use of medical information (e.g., contrast of inconsistencies between what the patient expresses and what is observed in medical tests or clinical history) was the most commonly used method to evaluate the risk of feigning/malingering (72.9%). Furthermore, obtaining an external or internal gain (33.3%) and inconsistencies between clinical presentation and findings (31.3%) were the evidence that raise their suspicion of feigning/malingering the most. The vast majority of medical professionals (81.3%) think that they have few means to detect feigning/malingering with certainty, and that it would be necessary to develop new methods, techniques, or tools to detect feigning/malingering (95.5).

**Table 5:** Experience of forensic physicians (*N* = 48)

	*n*	%
*11. Risk of feigning/malingering in your daily professional practice – annual estimate*		
Moderate frequency (between 20 and 40% of cases)	25	52.1%
High frequency (above 40%)	15	31.3%
Frequency lower than 20% of cases	8	16.7%
*12. Symptoms or diseases most commonly feigned by YOUR OWN patients/clients*		
Cervical damage	28	58.3%
General pain	8	16.7%
Fibromyalgia	4	8.3%
Depressive symptoms	3	6.3%
Lumbar pain	3	6.3%
Traumatic brain injury	2	4.2%
*13. Most common reasons for YOUR patients to feign*		
Disability or sick pay provided by a private insurance company	17	35.4%
Social security benefits	10	20.8%
Financial compensation	9	18.8%
Sick leave from work or education	5	10.4%
Securing benefits in a legal proceeding	4	8.3%
Obtaining medication	3	6.3%
*14. Method(s) used to detect the risk of feigning/malingering – possibility to choose more than one response*		
Use of medical information (e.g., contrast of inconsistencies between what the patient expresses and what is observed in medical tests or clinical history)	35	72.9%
Professional experience complementary to the use of specialized psychometric instruments	7	14.6%
Psychometric screening instruments to assess malingering (e.g., Structured Inventory of Malingered Symptomatology)	6	12.5%
*15. Data that raise suspicion of the existence of a risk of feigning/malingering*		
Obtaining an external or internal gain	16	33.3%
Inconsistency between clinical presentation and results	15	31.3%
Hostility, lack of collaboration, defensiveness	10	20.8%
Disproportionate or magnified symptom expression	5	10.4%
Lack of response to treatment	2	4.2%
*16. Adequacy of means to assess the risk of feigning/malingering*		
I have few means to detect it with certainty	39	81.3%
I have the means to detect it, but I do not believe that they are sufficient to detect it with an appropriate level of certainty	7	14.6%
I have sufficient means to detect it with certainty	2	4.2%
*17. It would be of interest to develop new methods, techniques or instruments to assess the risk of feigning/malingering*		
Yes, I think it would be necessary for my discipline	46	95.8%

## Discussion

In this study, we investigated the estimated base rates of feigning in Spain by using different methodologies. Our results indicate that approximately two-thirds of the participants reported knowing one or more people who had feigned or were currently feigning symptoms or an illness, and one-third reported that they themselves have feigned symptoms at some point in the past. These results align well with previous research. For instance, Dandachi-FitzGerald et al. (2020) found in a sample of 404 Dutch non-clinical participants that 59% knew a person who feigned or had feigned symptoms, and 34% admitted to having feigned symptoms. Merten and Giger (2018) observed, in a sample of 39 Swiss participants from the general population, a rate of 41% claiming to have feigned symptoms in the past. Furthermore, Schlicht and Merten (2014) found in a small pilot study with 20 German participants that 73% of them claimed to have either feigned symptoms themselves or to know people in their personal or professional environment who had.

The three symptom domains perceived as most frequently targeted were migraines or headaches, cervical damage, and depression. When comparing these results for each of the groups, it can be seen that these symptoms mostly coincide, except for the order of frequency. The high frequency of assumed feigning cervical damage is consistent with the findings of Santamaría et al. (2013), who observed in a sample of 161 Spanish physicians that the most frequent conditions of suspected feigning were whiplash injury, fibromyalgia, and chronic cervicalgia. In contrast, psychologists considered depressive symptoms, memory problems, anxiety, post-traumatic stress, and psychotic symptoms to be more relevant. The perception of feigned conditions seemed to depend, to a large extent, on the context of the professionals’ own work environment.

The results of our survey also support studies such as those conducted by Capilla Ramírez and González Ordi (2012), Puente-López et al. (2020), Represas et al. (2020), and Santamaría et al. (2013), who argue that, in Spain, whiplash injury and, in general, those conditions related to chronic pain are the most problematic and controversial, followed by conditions related to anxiety and depression. Symptoms such as pain are difficult to objectify with currently available diagnostic methods and cannot be properly quantified. Diagnoses of these types of conditions are usually based on the patients’ report of symptoms, without adequate objective criteria. The results obtained in the group of physicians to questions 14 (What method(s) do you typically use to assess malingering risk?), 16 (Please rate the extent to which you believe you have sufficient means to achieve reliable malingering detection), and 17 (Do you think that, at present, it would be of interest to develop new methods, techniques or instruments for the detection of malingering in your professional area?) show that they were aware of the absence of objective methods.

Most of the physicians indicated that they used medical information to detect possible feigning/malingering, while 12 to 14% responded that they used special psychometric instruments. However, the vast majority of experts admitted that they did not have sufficient means to be able to assess, with sufficient confidence, possible feigning or malingering. They stressed the need to develop new methods, techniques, or instruments for this purpose. Our findings are also consistent with an underuse of psychometric measures (particularly SVTs and PVTs) by physicians in Spanish medicolegal contexts (only 6% of our sample used validity tests), as previously described by Santamaría et al. (2013), which found that none of their participants mentioned the use of SVTs. It is not common for non-psychologists to use validity tests as they are usually not trained in their use. Rather, they oftentimes rely on clinical judgment, which is far from an accurate method for detecting malingering (Sweet et al., 2021). Hence, more reliable methods should be used. In an interdisciplinary approach, cooperation with psychologists should be sought in order to employ empirically founded and well-developed methods of validity assessment. This would, in return, increase the credibility and trustworthiness of problematic illness presentations onto the level of evidence-based approaches.

Unlike medical practitioners, forensic psychologists were more familiar with psychometric instruments, such as the Structured Inventory of Malingered Symptomatology (SIMS; Widows & Smith, 2005) and other instruments with embedded validity scales, such as the Minnesota Multiphasic Personality Inventory (MMPI-2-RF; Ben-Porath & Tellegen, 2008). Eighty percent of our psychologist sample claimed reported to use them regularly. In comparison to studies from other countries, this is a high percentage. For example, Cartwright et al. (2019) found for their UK sample that only 20% of medicolegal psychologists used validity tests, Yoxall et al. (2010) found that Australian medicolegal psychologists did not use them routinely, and Giromini et al. (2022) also found that only 13.2% of surveyed Italian psychologists reported using stand-alone SVTs or PVTs but “more than 60% spontaneously mentioned relying on these or similar kinds of validity checks, when inquired about their SVA routines” (p.8). One possible explanation for our results is that Spanish forensic psychology bibliography gives great importance to what authors have called the “differential diagnosis of malingering” (the term “feigning” is hardly ever used), and establishes that its assessment is “mandatory” in the forensic context (Arce, 2017; Gancedo et al., 2021), so some measure for its “control” is usually included. Specifically, the use of the SIMS, the MMPI-2, or both is highly frequent (e.g., Horcajo et al., 2017; Pallaro & González-Trijueque, 2009 or Vázquez & Catalán, 2008), since they are considered to be measures “validated for the forensic context” (Gancedo et al., 2021, González-Ordi et al., 2012; Sierra et al., 2006).

However, in Spain, the assessment of malingering is usually explained in a superficial and outdated manner, and there is currently no best practice guide that includes the latest findings on the subject. Also, although several instruments most widely used at an international scale have been validated for the Spanish language, such as the SIMS or the MMPI-2-RF, to date, only a limited number of tools are available in that language. It is possible that, for this reason, the vast majority of psychologists felt that they did not have sufficient means to reliably assess potential feigning and that the development of more methods or instruments is needed for this purpose. It is a challenge to remain on par with international developments. In this regard, ongoing efforts are directed toward Spanish-language adaptations of the Self-Report Symptom Inventory (SRSI; Merten et al., 2016), the M-FAST (Miller, 2001) and the Inventory of Problems-29 (IOP-29; Viglione & Giromini, 2020).

The professionals in the two groups were also asked about the estimated percentage of risk of feigning or malingering in their professional practice. For most of them, an estimation of feigning of between 20 and 40% of all cases was obtained. As indicated in the introduction, the great heterogeneity of published studies means that estimates of the prevalence of feigning differ greatly (Merten & Merckelbach, 2020). Our findings are similar to those of Mittenberg et al. (2002), Greve et al. (2009), and Chafetz (2011), who found a base rate in NA samples of 7 to 31%, 32.5%, 36%, and 38.5%, respectively. However, our estimates are lower than those of other studies, such as the 46% of Schroeder et al. (2021) from a sample of North America social security disability claimants and the 35 to 55% of Hall and Kalus (2021) from a sample of UK litigant population. Yet, our results are considerably higher than the 9.9% UK base rate estimated by expert psychologists (Cartwright et al., 2019), 5 to 10% base rate of Australian psychologists doing medicolegal work (Yoxall et al., 2010), and the estimated 13% among Australian neuropsychologists (Sullivan et al. 2006). Our base rate is also significantly lower than that identified by Santamaría et al. (2013) in a study with a similar Spanish sample. The medical forensic experts who participated in the Santamaría et al. study estimated the prevalence rate to be close to 50% in cases of chronic pain disorders. However, the experts considered whiplash injury to be the condition with the highest prevalence of feigning, which is consistent with what was reported by our forensic expert participants.

These prevalence estimation differences may be explained by the year in which the Santamaría et al. (2013) study was conducted. In 2015, the economic compensation system for suffering some kind of injury after a traffic accident, which is considered the main cause of whiplash injury (Represas et al., 2020), was modified with the Ley 35/2015, of September 22, on the reform of the system for the valuation of damages caused to persons in traffic accidents, which made it more difficult to obtain a compensation. Thus, before the introduction of the 2015 Law, feigning an injury such as a whiplash injury, and obtaining a significant external reward for it, was easier, which would increase the perceived risk of feigning of this type of conditions.

Overall, it seems that Spanish medicolegal practitioners perceive that they face a higher risk of feigning in their professional practice than in some other non-North American countries. This could be explained by the two issues discussed above. Firstly, pain-related conditions are considered “easy to fake, hard to objectify.” In particular, this relates to cervical pain conditions. Whiplash injury is commonly called “cuponazo cervical” in Spain, which is the equivalent of “a cervical lottery coupon.” Many medical professionals are skeptical when seeing whiplash patients. The fact that the participating psychologists consider cervical injury to be one of the conditions most frequently feigned, with practically no relationship with these patients, supports the hypothesis that they are perceived as high risk. Given that much of the work of the medical experts consulted is dealing with these types of conditions, it is likely that they perceive that they are exposed to a higher risk of feigning/malingering. On the other hand, the perception of prevalence offered by the participating psychologists may be explained by the high use of SVTs. In studies such as Cartwright et al. (2019), 20% of participating psychologists claimed to use SVTs in their professional practice and reported a prevalence of 9.9%. Given that the use of subjective clinical judgment is not an efficient method for symptom validity assessment (Sweet et al., 2021), it is possible that experts who rely on its use detect fewer cases, which would influence their perception of the occurrence of feigning and cause them to underestimate it. In addition to the high use of SVTs, the erroneous belief has been identified in Spain that SIMS is a “malingering test” and that a positive result is equivalent to malingering detection (Merten et al., 2013, 2021). This could increase the number of detected cases and alter the psychologist perception of the occurrence of the event, in this case overestimating it.

The motives that our participants considered most important for feigning were to obtain a sick leave from work or education, followed by obtaining social security benefits (such as disability or unemployment payment), and obtaining financial compensation, provided by a private insurance system. Again, our findings are consistent with those obtained by Dandachi-FitzGerald et al. (2020), in which participants listed sick leave as the main reason for feigning. However, these authors found that psychological motives (excusing a failure and seeking attention from others) ranked second and third among the top five motives for feigning, and they considered these psychological motives to be “determinants of everyday feigning” (p. 229). They also pointed out that many publications focused exclusively on external economic motives, which was an incomplete view. Although our findings pointed to economic motives as the main incentives for the presentation of feigned illness, we agree that psychological motives are of great importance in understanding why people feign illness.

Regarding the chosen symptoms to feign, our participants indicated that, if they were in a situation of feigning an illness, they mainly chose common symptoms, such as headache, stomachache, or common cold, or those related to neck, lower back, or general pain. The main reasons for this choice were that they were easy to feign, they had suffered from them before or that they claimed to know someone who had suffered from them before. These results are also consistent with what Dandachi-FitzGerald et al. (2020) and Dandachi-FitzGerald and Merckelbach (2013) found. As pain-related symptoms are the most difficult to objectify, hence, assess, except in very specific cases (see Greve et al., 2012), these results indicate that even novel psychological methods of validity assessment may fail to aid the detection of feigning in a substantial proportion of real-world cases.

We also asked psychology undergraduates, who had already completed their courses, about the preparation they received on the subject. Three quarters of the participants in this group received specific university education on malingering, whereas other concepts of importance in the area, such as feigning, distorted symptom presentations, symptom validity, or response style distortion, have been the subject of much rarer teaching. Among the instruments, the SIMS and the TOMM, and two protocols developed in Spain (Sistema de Evaluacion Global, SEG; Arce & Fariña, 2004, 2005 and Entrevista Clínico-forense; Arce & Fariña, 2001) were named. Half of the participants considered their education to be insufficient. It should also be taken into account that not all Spanish study programs include subjects related to forensic psychology, so receiving such education will depend on taking a master’s degree in the area of legal and forensic specialization, and that this master’s degree includes it in its contents.

About 10 years ago, the state of the art of symptom and performance validity assessment in the country was described as “quality seeds [being] available to get a good harvest in Spain (but) further research and educational effort will be necessary for establishing sound practice guidelines and protocols, both for researchers and professionals” (Merten et al., 2013, p.134). At present, there are more open questions than answers, and there is still a need for such an effort, especially at the research level. As stated in a recent update of the 2013 review (Merten et al., 2021), there were no guidelines for validity testing in Spain, a problem that still persists. Also, more psychometric instruments need to be validated, and efforts should be made to provide a more complete and comprehensive education on the subject, including the use of current terminology and knowledge about the most recent developments.

The results described above should be interpreted bearing in mind the following limitations: First, the method used was a survey, which may be affected by recall bias (Uiterwijk et al., 2021). Second, it should be noted that requesting rough prevalence estimates from professionals is a process that may be affected by the effect of a priori probabilities, or the tendency to overestimate the prevalence of relevant conditions (Santamaría et al., 2013). Consequently, the data should not be mistaken as being the true base rates of problematic illness presentations in Spain, but give an approximate picture of their occurrence. As Santamaría et al. (2013) point out, such survey results should be considered “rather as an approach to the professional (and non-professional) point of view and the relevance they attach to a problem with a strong impact on the economic resources of healthcare systems” (p. 146). Third, the experts were not specifically asked what information they relied on to make their estimates. Given that only 6% of the physicians used symptom validity tests, it is possible that practitioners’ beliefs about prevalence rates were based solely on clinical judgment, making their estimates less reliable. Fourth, like Dandachi-FitzGerald et al. (2020), we used proxy respondents because of the subject matter. The main consequence of this is that they may be less accurate in determining the underlying motivations for faking. Fifth and last, because the topic chosen may be uncomfortable, it is difficult to rule out entirely the possibility that some bias in the participants’ response style, such as positive impression management or self-deception, may have occurred. However, the survey was completely anonymous and the method of proxy respondents used decreases the risk of this occurring.

Surveys of problematic illness presentations appear to play an important role in validity research, starting with that seminal work by Mittenberg et al. (2002). They are able to highlight the importance of validity assessment in different contexts and different patient populations. Since Mittenberg et al.’s report, a greater number of surveys from different parts of the world have been published. From a cross-cultural perspective (e.g., Nijdam-Jones & Rosenfeld, 2017), results cannot be generalized across national, linguistic, and cultural boundaries. Next to the linguistic and cultural differences often discussed in the literature, concepts of illness, the national organization of welfare programs, and social security legislation play an important role. For instance, differences in the ethical dimensions of how issues, such as social welfare abuse or fraudulent insurance claims, are accepted or not in the general population are exceptionally impactful. In this regard, the results from the USA and Spain cannot be compared directly. A systematic and evidence-based approach to cross-cultural differences in problematic illness presentations has yet to be developed, but surveys as this one may provide valuable pieces of a more comprehensive picture.

## Data Availability

The data that support the findings of this study are available from the corresponding author upon reasonable request.
